# Hypertonic Saline Dextran Ameliorates Organ Damage in Beagle Hemorrhagic Shock

**DOI:** 10.1371/journal.pone.0136012

**Published:** 2015-08-28

**Authors:** Jing-xiang Zhao, Bo Wang, Guo-xing You, Ying Wang, Gan Chen, Quan Wang, Xi-gang Zhang, Lian Zhao, Hong Zhou, Yue-zhong He

**Affiliations:** 1 Institute of Transfusion Medicine, Academy of Military Medical Sciences, No. 27th Taiping Road, HaiDian, Beijing, China; 2 Emergency department, Chinese People’s Liberation Army 307 hospital, No. 8th Dongda Street, Fengtai, Beijing, China; 3 Science and Technology department, Academy of Military Medical Sciences, No. 27th Taiping Road, HaiDian, Beijing, China; King Abdullah International Medical Research Center, SAUDI ARABIA

## Abstract

**Objective:**

The goal of this study was to investigate the effect of hypertonic saline with 6% Dextran-70 (HSD) resuscitation on organ damage and the resuscitation efficiency of the combination of HSD and lactated ringers (LR) in a model of hemorrhage shock in dogs.

**Methods:**

Beagles were bled to hold their mean arterial pressure (MAP) at 50±5 mmHg for 1 h. After hemorrhage, beagles were divided into three groups (n = 7) to receive pre-hospital resuscitation for 1 h (R1): HSD (4 ml/kg), LR (40 ml/kg), and HSD+LR (a combination of 4 ml/kg HSD and 40 ml/kg LR). Next, LR was transfused into all groups as in-hospital resuscitation (R2). After two hours of observation (R3), autologous blood was transfused. Hemodynamic responses and systemic oxygenation were measured at predetermined phases. Three days after resuscitation, the animals were sacrificed and tissues including kidney, lung, liver and intestinal were obtained for pathological analysis.

**Results:**

Although the initial resuscitation with HSD was shown to be faster than LR with regard to an ascending MAP, the HSD group showed a similar hemodynamic performance compared to the LR group throughout the experiment. Compared with the LR group, the systemic oxygenation performance in the HSD group was similar but showed a lower venous-to-arterial CO_2_ gradient (Pv-aCO_2_) at R3 (p < 0.05). Additionally, the histology score of the kidneys, lungs and liver were significantly lower in the HSD group than in the LR group (p < 0.05). The HSD+LR group showed a superior hemodynamic response but higher extravascular lung water (EVLW) and lower arterial oxygen tension (PaO_2_) than the other groups (p < 0.05). The HSD+LR group showed a marginally improved systemic oxygenation performance and lower histology score than other groups.

**Conclusions:**

Resuscitation after hemorrhagic shock with a bolus of HSD showed a similar hemodynamic response compared with LR at ten times the volume of HSD, but HSD showed superior efficacy in organ protection. Our findings suggest that resuscitation with the combination of HSD and LR in the pre-hospital setting is an effective treatment.

## Introduction

Hemorrhagic shock is the leading cause of morbidity and mortality in trauma and military casualties. Fluid resuscitation is one of the first essential treatments of severe hemorrhagic shock and may have significant effects on both early and late outcomes. Hypertonic saline dextran (HSD, 7.5% NaCl in 6% Dextran) has been confirmed to be a highly effective resuscitation solution for the treatment of life-threatening hemorrhage [1–5]. Administration of HSD can rapidly restore intravascular volume and tissue perfusion, enhance microcirculatory flow, alleviate inflammatory response [6], and ultimately leading to an improvement in survival. Additionally, HSD has advantages when used for pre-hospital fluid resuscitation due to its high volume effect, limited edema formation, and marked reduction of baggage load for rescue forces [5, 7].

As with other artificial colloid such as hydroxyethyl starch and gelatin, HSD was also reported pitfalls including anaphylactoid reactions, worsening of coagulopathy and renal function. However, data from animal and clinical trial suggested that HSD caused minimal risk after infusion of the proposed therapeutic dose of 4ml/kg [8,9]. Compared with 0.9% NaCl (NS), although initial resuscitation with a single bolus of HSD worsen hypo-coagulability and hyper-fibrinolysis [10], but did not reduce the survival of patient [11]. Moreover, small volume resuscitation fluids continue to be of interest to the military and limited volume resuscitation is becoming more common in the treatment of hemorrhage in the civilian community. And HSD has been suggested as small volume expander for early hemorrhagic shock. Therefore, HSD was still a popular resuscitation fluid in many countries.

Although HSD is superior as a pre-hospital treatment, it is not commonly used in civilian ambulance rescues. The ambulance services’ clinical practice guidelines in many countries only suggest lactated ringers (LR) and NS as the resuscitation fluids to correct hypovolemia [12]. The possible reason for this may be that the advantage of HSD over LR for improving survival from hemorrhagic shock remains controversial in clinical studies. In the studies of Mattox KL and Wade CE, the clinical trials that compared crystalloid infusion (i.e., standard of care) with HSD, followed by crystalloids, for the pre-hospital resuscitation of hypotensive trauma patients showed that resuscitation with HSD produced better survival in victims with penetrating injuries requiring surgery [13, 14]. Conversely, Bulger’s study showed that among injured patients with hypovolemic shock, the initial resuscitation fluid treatment with HSD compared with NS did not result in superior 28-day survival [11]. It is well known that fluid resuscitation is the initial intervention for hemorrhagic shock, increasing survival time of casualties after leaving the hospital. While the definitive care, such as blood transfusion, operations etc., in emergency medical services (EMS) agencies is the typical essential therapy for later survival. In Bulger’s study, the patient was transported to the EMS quickly (i.e., within approximately 30 mins); This likely produced the results that the initial fluid resuscitation has less effect on the patient survival compared with the definitive treatment in EMS. Therefore, in the civilian setting in which transport times are short, the advantage of HSD may not be as clear. However, in remote or underdeveloped areas, casualties had to take prolonged transport periods to definitive treatment. So, fluid resuscitation therapy inevitably matters significantly, and survival could be affected by the initial transfusion of HSD and crystalloid infusion. Therefore, the superior efficacy of the use of HSD for the treatment of hemorrhagic hypotension should be studied in more detail, particularly with regard to rescuing casualties who require longer evacuation times.

The initial goal of fluid resuscitation after hemorrhagic shock is to restore hemodynamic stabilization, but the ultimate goal is to correct tissue hypoxia and to alleviate organ damage. Previous animal studies of HSD resuscitation compared with LR primarily focused on physiologic outcomes and hemodynamic effects, indicating similar hemodynamic benefits between an initial bolus of HSD and LR with eight to ten times the volume of HSD [7, 15–17]. However, data regarding the influence of HSD resuscitation on organ dysfunction are rare. Therefore, to evaluate the effect of HSD resuscitation, organ damage should be studied further.

In previous clinical trials, the administration of HSD has been followed by conventional fluids such as lactated Ringer’s solution or normal saline [18]. Because colloids and crystalloids have different effects on a range of important physiological parameters, some studies state that the combination of colloid with crystalloid for fluid resuscitation may be beneficial [19]. Therefore, we tested the hypothesis that the combination of HSD and LR in the pre-hospital setting would yield superior resuscitation efficacy from Hemorrhagic shock than LR alone in the setting of an ambulance rescue.

Therefore, the purpose of this study was to compare the effects of resuscitation using HSD or LR on organ damage in the case of similar hemodynamic efficacy; This was accomplished using a model of hemorrhage shock in dogs. We also attempted to determine the advantages and disadvantages of the combination of HSD and LR in pre-hospital resuscitation.

## Materials and Methods

Experimental procedures were approved by the Animal Experimental Ethics Committee of the Institute of Transfusion Medicine, Academy of Military Medical Sciences. The research adhered to the guidelines for the care and use of laboratory animals, and the experimental animal population was treated humanely. All surgery was performed under pentobarbital anesthesia. Every effort was made to minimize any suffering of the animals used in this study.

### Animal Preparation and Instrumentation

Twenty-one adult male beagles, weighing between 8 and 9 kg, were used in this study. The animals were fed standard dog chow and were given water ad libitum in a divisional kennel. Each animal was anesthetized with pentobarbital (0.03 g/kg, iv) while maintaining spontaneous breathing and underwent sterile surgery. The femoral arteries, right femoral veins and right external jugular veins were isolated and cannulated with catheters. A 4F PiCCO catheter (PV2014L08N, Pulsion Medical system, Munich, Germany) was inserted into the left femoral artery to measure the blood pressure and continuous cardiac output using a pulse contour analysis computer (PiCCO, Drager Medical Systems, Inc. USA) monitor, and a 4F central venous catheter was placed through the external jugular vein to measure the central venous pressure (CVP) and to sample the mixed venous blood. The right femoral artery was used to produce bleeding using an infusion pump and to sample the arterial blood. The right femoral vein was used to administer fluids.

After the end of resuscitation, supplemental doses of sodium pentobarbital (0.005 g/kg, iv) were given per hour to keep the animals anesthetized until the end of surgery.

### Experimental Protocol

**Fig 1:**
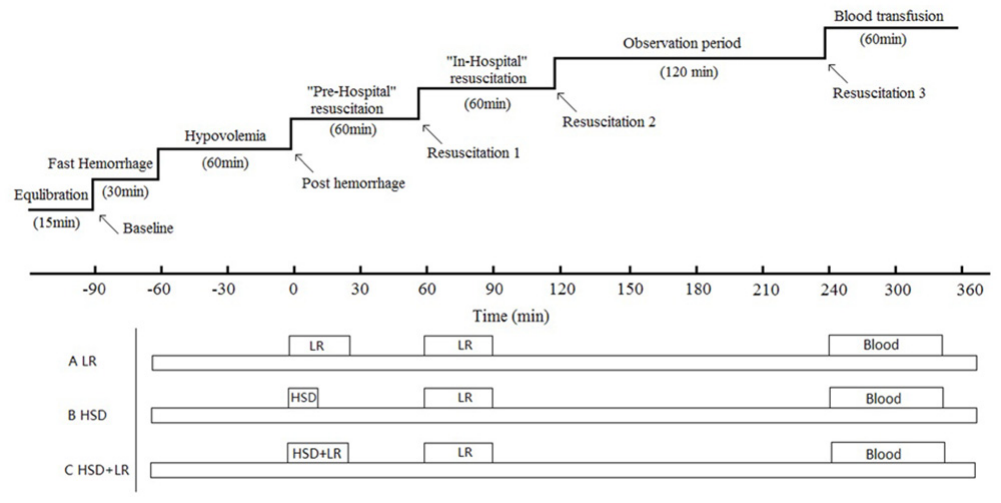
Experimental protocol. Following an equilibration period, baseline (BL) measurements of all parameters were recorded, and subsequent blood was rapidly withdrawn until a mean arterial pressure of approximately 50±5 mm Hg was reached and maintained for another 60 minutes (hypovolemia period). All measurements were then recorded (post-hemorrhage, PH, time = 0 min), and the animals were separately resuscitated with HSD, LR or HSD+LR over 60 minutes (Resuscitation1, R1, time = 60 min). Then, during the in-hospital resuscitation phase, LR were started as a transfusion for all animals until the MAP reached 85% of the baseline value (Resuscitation 2, R2, time = 120 min). After a two-hour observation period (Resuscitation 3, R3, time = 240 min), all animals received blood transfusions (70% of their blood loss) so that they would survive for three days of observation. Then, the dogs were sacrificed with an IV bolus injection of pentobarbital, and their tissues including their kidneys, lungs, liver and intestines were obtained for pathological analysis.

Fig 1. shows the experimental protocol. Following the operation and instrumentation, the dogs were allowed to stabilize for 15 minutes (equilibration period). After baseline measurements were taken, the subjects were rapidly hemorrhaged (20 ml/min) from the femoral artery until the mean arterial pressure (MAP) reached 45 mmHg (fast hemorrhage phase); hemorrhaging was continued as required to maintain the MAP at 50±5 mm Hg for 60 minutes (hypovolemia phase). After hemorrhaging, the experimental time was set to zero (post-hemorrhage, PH, time = 0), and the beagles were divided into three groups to receive pre-hospital resuscitation for one hour (Resuscitation1, R1, time = 60 min): (1) LR (lactated Ringers, 40 ml/kg, China Shijiazhuang Pharmaceutical Co., Led., Shijiazhuang, China); (2) a bolus of HSD (NaCl 7.5%-Dextran70 6%, 4 ml/kg, Beijing Double-Crane Pharmaceutical Co., Led., Beijing, China); (3) HSD+LR, which was a combination of 4 ml/kg HSD and 40 ml/kg LR where HSD was injected first, followed by LR. HSD was injected intravenously over a fixed interval of 2 min. Lactated ringers were intravenously infused at a constant rate of 20 ml/min. LR was then transfused for all groups in-hospital until the MAP reached 85% of the baseline value (Resuscitation2, R2, time = 120 min). Following two hours of observation (Resuscitation3, R3, time = 240 min), autologous blood (70% of shed blood) was transfused. Then, all animals were sewn up and taken back into their cages to awaken naturally. Anti-infective therapy for the dogs was implemented by injecting benzylpenicillin sodium (0.48 g, China Shijiazhuang Pharmaceutical Co., Led., Shijiazhuang, China) per day. After three days, the dogs were sacrificed with an IV bolus injection of pentobarbital, and their tissues including kidney, lung, liver and intestinal were obtained for pathological analysis.

### Measurements

#### Hemodynamic parameters

Using the PiCCO system, a 5 ml bolus of cold saline (0°C) was injected via a central venous catheter into the right atrium, and the thermodilution curve was recorded to measure the cardiac index (CO) by the arterial catheter. Using the PiCCO continuous cardiac output monitoring system, the mean arterial blood pressure (MAP), cardiac index (CI), systolic volume index(SVI), global end-diastolic index (GEDVI), total intrathoracic blood index (ITBI), systemic vascular resistance index (SVRI) and extravascular lung water (EVLW) were all determined. The pulmonary vascular permeability index (PVPI) was calculated by EVLW/PBV [20].

#### Blood gas and oxygen parameters

Arterial and mixed venous blood gas, including pH, PO_2_, PCO_2_, base excess (BE), lactate (Lac), hemoglobin (Hb), arterial oxygen saturation (SaO_2_) and central venous oxygen saturation (ScvO_2_), were all measured with a blood-gas analyzer (ABL90 FLEX, Radiometer Copenhagen, Denmark). Arterial and mixed venous contents (CaO_2_ and CvO_2_, respectively) were calculated as follows:

$$CaO_{2}=1.34\times \text{Hb}\times {\text{SaO}}_{2}+0.0031\times {\text{PaO}}_{2}$$

$$CvO_{2}=1.34\times \text{Hb}\times SvO_{2}+0.0031\times PvO_{2}$$

Systemic oxygen transport and uptake (DO_2_ and VO_2_, respectively) were estimated as:

$$DO_{2}=\text{Cl}\times CaO_{2}\times 10$$

$$\;VO_{2}=\text{Cl}\times CvO_{2}\times 10$$

The venous-to-arterial CO_2_ gradient (P_(v-a)_CO_2_) was calculated as the difference between PvCO_2_ and PaCO_2_ and was simultaneously and respectively obtained from the central mixed venous blood and arterial blood samples.

#### Tissue injury

The total urine output during the anesthetic period was measured. Plasma levels of urea nitrogen (BUN) and alanine aminotransferase (AST) were evaluated using a commercial kit (Leadman Group Co., Ltd. Beijing, China) and HITACHI 7020 auto analyzer (HITACHI, Tokyo, Japan).

After three days of resuscitation, the dogs were sacrificed with an IV bolus injection of pentobarbital. The kidneys, lungs, liver and intestines were excised, fixed in formalin, microtomized, and stained in hematoxylin and eosin (H&E) and Periodic Acid-Schiff to observe histological changes.

Kidney damage was scored by grading any glomerular, tubular, and interstitial change, based on a previous study [21]. The sum of the partial scores resulted in a final grade from 0 to 9.

The lung parenchyma from each animal was graded on a scale from 0 to 3 (0, none; 1, slight; 2, moderate; 3, severe) for pulmonary interstitial inflammation, alveolar septal fibrosis, fibrin exudation, and bronchial epithelial lesions. The sum of the partial scores resulted in a final grade from 0 to 12.

Histological changes in the liver were assessed in the hepatic lobule and portal area by sinusoidal congestion and cytoplasmic vacuolization, inflammatory cell infiltration, and necrosis of parenchymal cells and scored on a 4-point scale (0, none; 1, slight; 2, moderate; 3, severe). The sum of the partial scores resulted in a final grade from 0 to 9.

The intestines were graded on a scale from 0 to 3 (0, none; 1, slight; 2, moderate; 3, severe) for interstitial inflammation and intestinal mucosa epithelial cell necrosis. The sum of the partial scores resulted in a final grade from 0 to 6.

### Statistical Analysis

Data were presented as means ± standard deviations (SD). The hemodynamics parameters and systemic oxygenation were analyzed using a two-way analysis of variance (ANOVA), followed by a Student-Newman-Keuls test as deemed appropriate. The differences in tissue injury between the groups were determined using a Student-Newman-Keuls one-way analysis of variance. When the normality and homogeneity of variance assumptions were not satisfied, a non-parametric Kruskal-Wallis test was applied. The significance level was set to *p*< 0.05. A commercial software package (SAS Institute Inc., Cary, NC, USA) was used for data analysis.

## Results

The average blood volume loss was 53±3 ml/kg, which corresponded to approximately 62%±4% of the estimated circulating blood (85 ml/kg) volume; There were no significant differences among the groups (p>0.05). All animals survived to the study’s end.

### Hemodynamics

**Fig 2:**
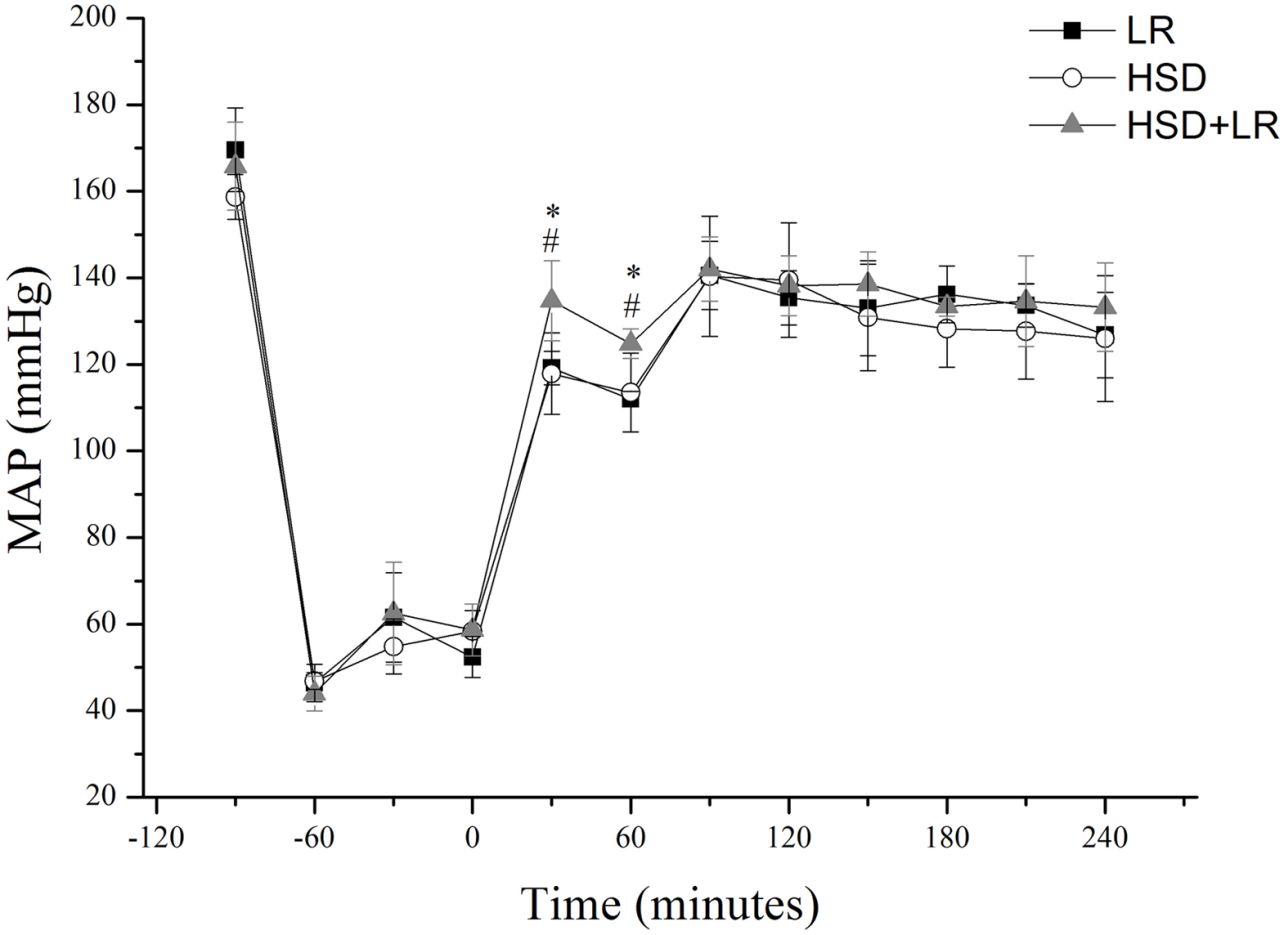
MAP in the LR, HSD and HSD+LR groups, measured at different time intervals. Pre-hospital resuscitation (0-60min) from hemorrhagic shock respectively with (1) 40ml/kg lactated ringers at a constant rate of 20 ml/min (LR group), (2) a bolus of HSD (4ml/kg) over a fixed interval of 2 min (HSD group), or (3) an initial bolus of HSD (4ml/kg) with a continued infusion of 40ml/kg Lactated Ringer (HSD+LR group). Subsequently all groups were injected with lactated ringers in in-hospital (60-90min) until the MAP reached 85% of the baseline level. *P<0.05 vs LR (*black square*); ^#^P<0.05 vs HSD (*open circle*); ^&^P<0.05 vs HSD+LR (*shaded triangle*). n = 7 for all groups.

All groups were equivalent at baseline and showed a similar hemodynamic response to hemorrhage. After pre-hospital resuscitation, the MAP increased progressively in all groups, and the MAP value in the HSD+LR group increased more significantly than that in the HSD or LR groups (p<0.05). Following the in-hospital infusion of LR, the MAP achieved 85% of baseline level in all groups. The MAP values did not show significant differences among the groups throughout the observation period (p>0.05) (Fig 2).

**Fig 3:**
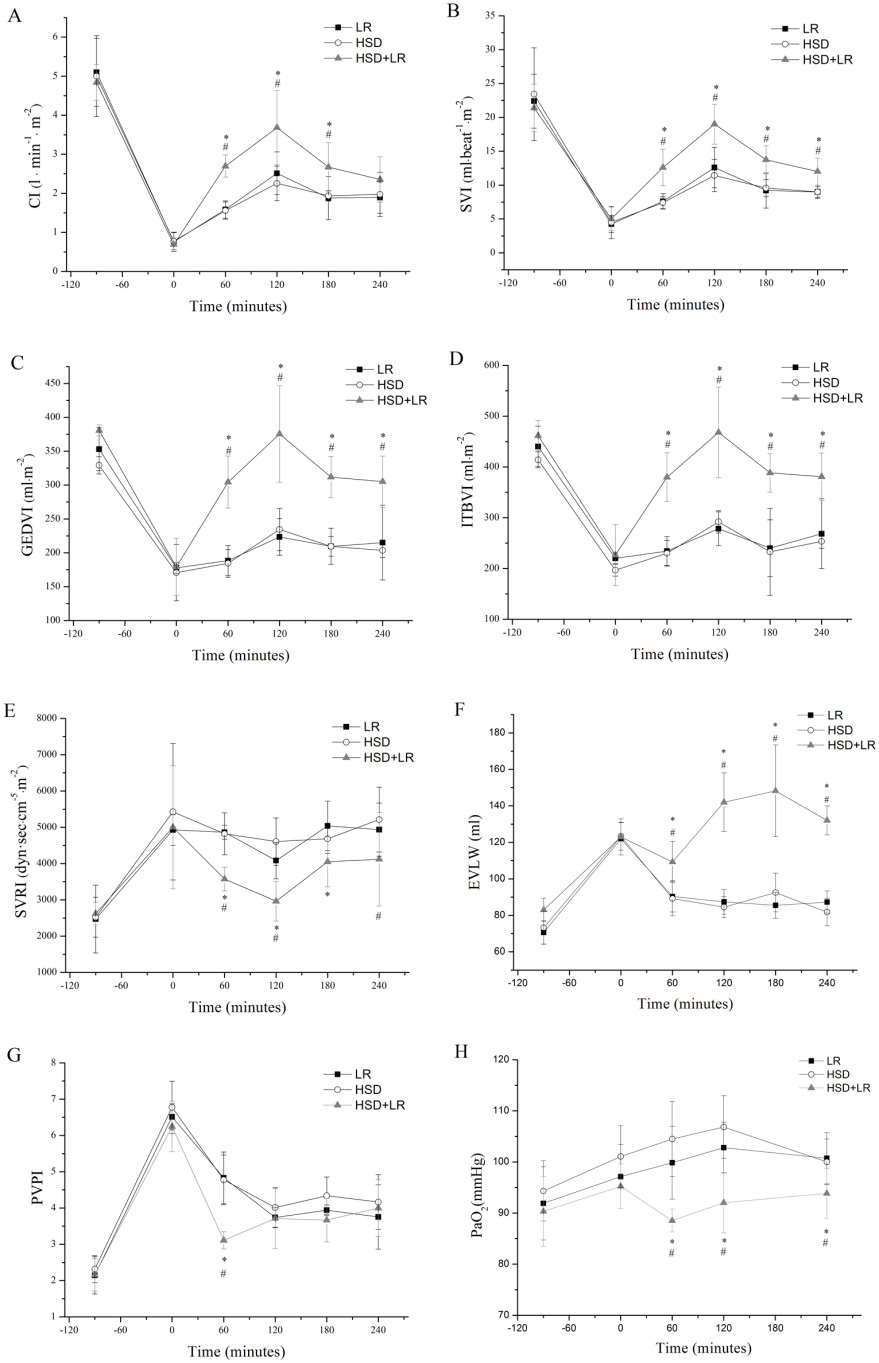
Hemodynamic parameters changes after hemorrhagic shock and resuscitation. Pre-hospital resuscitation (0-60min) from hemorrhagic shock respectively with (1) 40ml/kg lactated ringers at a constant rate of 20 ml/min (LR group), (2) a bolus of HSD (4ml/kg) over a fixed interval of 2 min (HSD group), or (3) an initial bolus of HSD (4ml/kg) with a continued infusion of 40ml/kg Lactated Ringer (HSD+LR group). Subsequently all groups were injected with lactated ringers in in-hospital (60-90min) until the MAP reached 85% of the baseline level. (A) cardiac index (CI). (B) systolic volume index (SVI). (C) global end-diastolic index (GEDVI). (D) total intrathoracic blood index (ITBI). (E) systemic vascular resistance index (SVRI). (F) extravascular lung water (EVLW) (G) pulmonary vascular permeability index (PVPI). (H) arterial oxygen tension (PaO_2_). *P<0.05 vs LR (*black square*); ^#^P<0.05 vs HSD (*open circle*); ^&^P<0.05 vs HSD+LR (*shaded triangle*). n = 7 for all groups.

**Fig 4:**
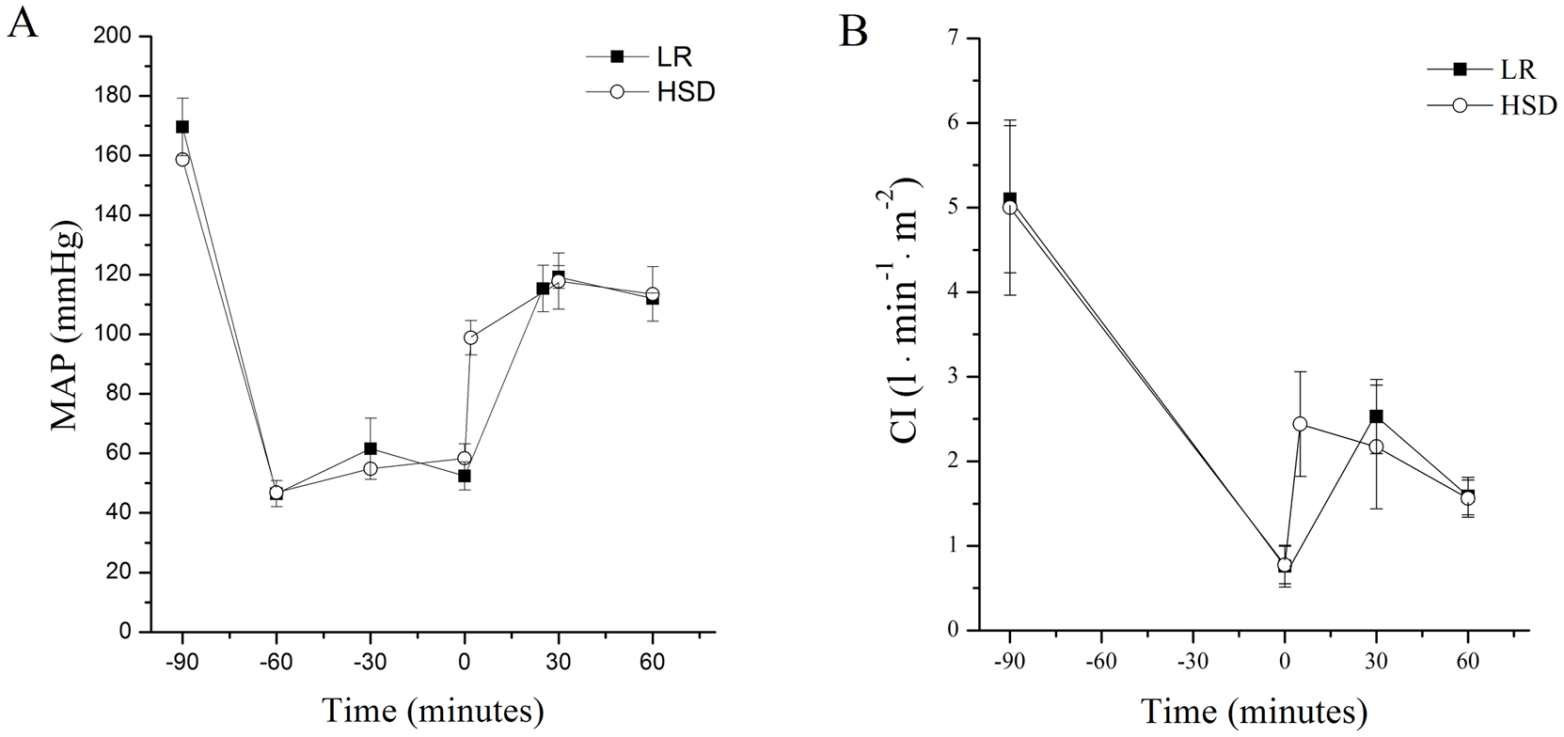
The changes of MAP and CI in the LR and HSD group during the pre-hospital period. (A) mean arterial pressure (MAP). (B) cardiac index (CI). A bolus of HSD (4ml/kg) was injected intravenously over a fixed interval of 2 min (HSD group). While, 40ml/kg Lactated ringers were intravenously infused at a constant rate of 20 ml/min (LR group). Therefore, the HSD infusion was completed more quickly and thus produced a faster increase in the MAP and CI than those with the LR infusion. *P<0.05 vs LR (*black square*); ^#^P<0.05 vs HSD (*open circle*). n = 7 for all groups.

Each of the groups showed an increase in cardiac output and a decrease in the systemic vascular resistance in response to resuscitation. The HSD+LR group showed a significantly higher CI, SVI, GEDVI, ITBI, and lower SVRI than the other groups throughout the resuscitation and observation periods (Fig 3A, 3B, 3C, 3D and 3E). In the HSD group, a bolus of 4 ml/kg HSD was injected intravenously over a fixed interval of 2 min. While, in the LR group, 40ml/kg LR were intravenously infused for about 20min in a constant rate of 20 ml/min. Therefore, the HSD infusion was completed more quickly and thus produced a faster increase in the MAP and CI than those with the LR infusion. However, the HSD group and the LR group showed a similar hemodynamic performance after the end of resuscitation (Fig 4).

Compared to the HSD group and LR group, the HSD+LR group showed a greater EVLW, a lower PVPI in the pre-hospital period (*P*<0.05) and a similar PVPI in the in-hospital period (*P*>0.05) (Fig 3F and 3G); these suggested that the higher amount of lung water in the HSD+LR group was induced by hydrostatic action [19]. Consistently, the arterial oxygen tension (PaO_2_) in the HSD+LR groups was also found to be below that in the other groups (Fig 3H). Generally, the arterial oxygen tension (PaO_2_) showed a tendency of increase during hemorrhage shock period. This elevation occurred due to hyperventilation, trying to compensate for the decrease in oxygen delivery to the tissues [22]. In this study, the PaO_2_ value increased in all animals after hemorrhage. However, the PaO_2_ value showed a similar increase in HSD and LR group during the initial resuscitation period, but presented a decrease tendency in the HSD+LR group. The changes of PaO_2_ in the HSD+LR group may be due to the combined functions caused by compensatory hyperventilation and the harmful effects of lung water on the ventilation function of lung.

### Systemic oxygenation

**Fig 5:**
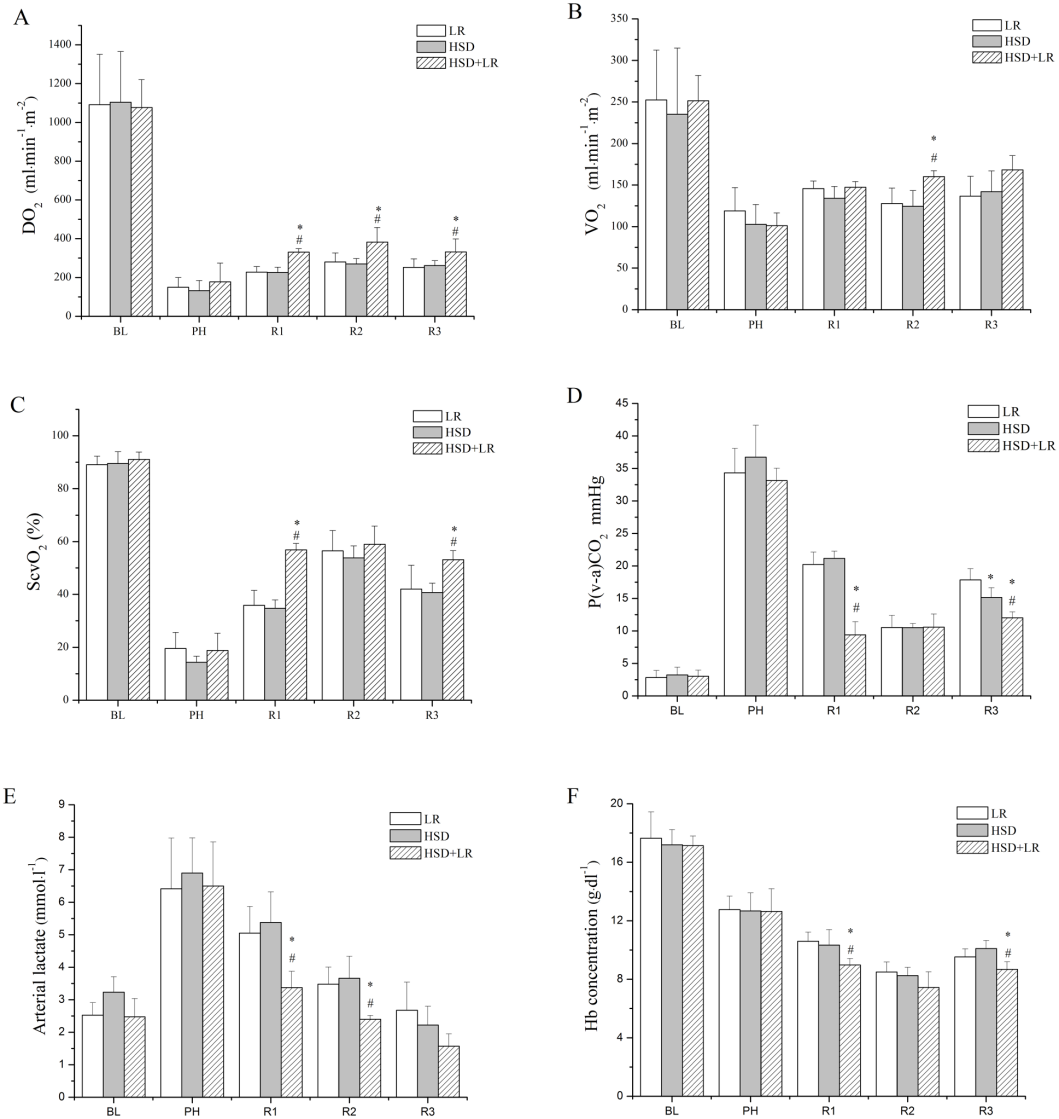
Systemic oxygenation parameters in the LR, HSD and HSD+LR groups presented at baseline (BL, time = -90 min), end of controlled bleeding (post-hemorrhage, PH, time = 0 min), end of pre-hospital resuscitation (Resuscitation1, R1, time = 60 min), end of in-hospital resuscitation (Resuscitation2, R2, time = 120 min), and 3 h after in-hospital resuscitation (Resuscitation3, R3, time = 240 min). (A) Systemic oxygen transport (DO_2_). (B) Systemic oxygen uptake (VO_2_). (C) Central venous oxygen saturation (ScvO2). (D) Venous-to-arterial CO_2_ gradient (Pv-aCO_2_). (E) Arterial lactate (Lac). (F) Hb concentration. *P<0.05 vs LR; ^#^P<0.05 vs HSD. n = 7 for all groups.

Hemorrhage also caused marked effects on the systemic oxygenation variables with no significant differences among the groups (p>0.05) (Fig 5). Signs of tissue hypoperfusion were evident. In all groups, there was a significant decrease of DO_2_, VO_2_, and ScvO_2_ and an increase in P(v-a)CO_2_ and arterial lactate.

After fluid resuscitation, the DO_2_ values were significantly higher in the HSD+LR groups than in the HSD or LR groups throughout the resuscitation and observation periods (Fig 5A). However, the VO_2_ values were higher in the HSD+LR groups than in the other groups only at R2 (time = 120 minutes) (Fig 5B). The ScvO_2_ values were significantly increased after fluid resuscitation and were higher in the HSD+LR groups than in the HSD and LR groups at R1 (time = 60 minutes) and R3 (time = 240 minutes) (Fig 5C). While, the P_(v-a)_CO_2_ and arterial lactate values were found to decrease after fluid resuscitation. The P_(v-a)_CO_2_ values were significantly lower in the HSD+LR group than in the HSD or LR groups (p<0.05) at R1 (time = 60 minutes) and R3 (time = 240 minutes). Additionally, the P_(v-a)_CO_2_ value in all groups consistently decreased after LR transfusion (R2) in-hospital but gradually increased at the end of the observation period (R3), while P_(v-a)_CO_2_ was significantly higher in the LR group than in the other groups (p<0.05) (Fig 5D). After resuscitation, the arterial lactate levels gradually decreased with time and were restored to the pre-hemorrhage levels by the end of the observation period (R3) (Fig 5E). Compared with the HSD and LR groups, the HSD+LR group showed significantly lower arterial lactate levels at R1 and R2 (p<0.005). The lactate value was also shown to be lower in the HSD+LR group at R3, but no significant differences were found among the groups (p>0.05). The Hb concentration was maintained below baseline levels after fluid resuscitation in all groups. The Hb concentration in the HSD+LR group was significantly lower than those in the LR and HSD groups at R1 and R3 (Fig 5F). Systemic oxygenation variables, including DO_2_, VO_2_, ScvO_2_, Hb and arterial lactate, were not significantly different between the HSD group and LR group throughout the experimental period (Fig 5).

### Tissue injury

**Fig 6:**
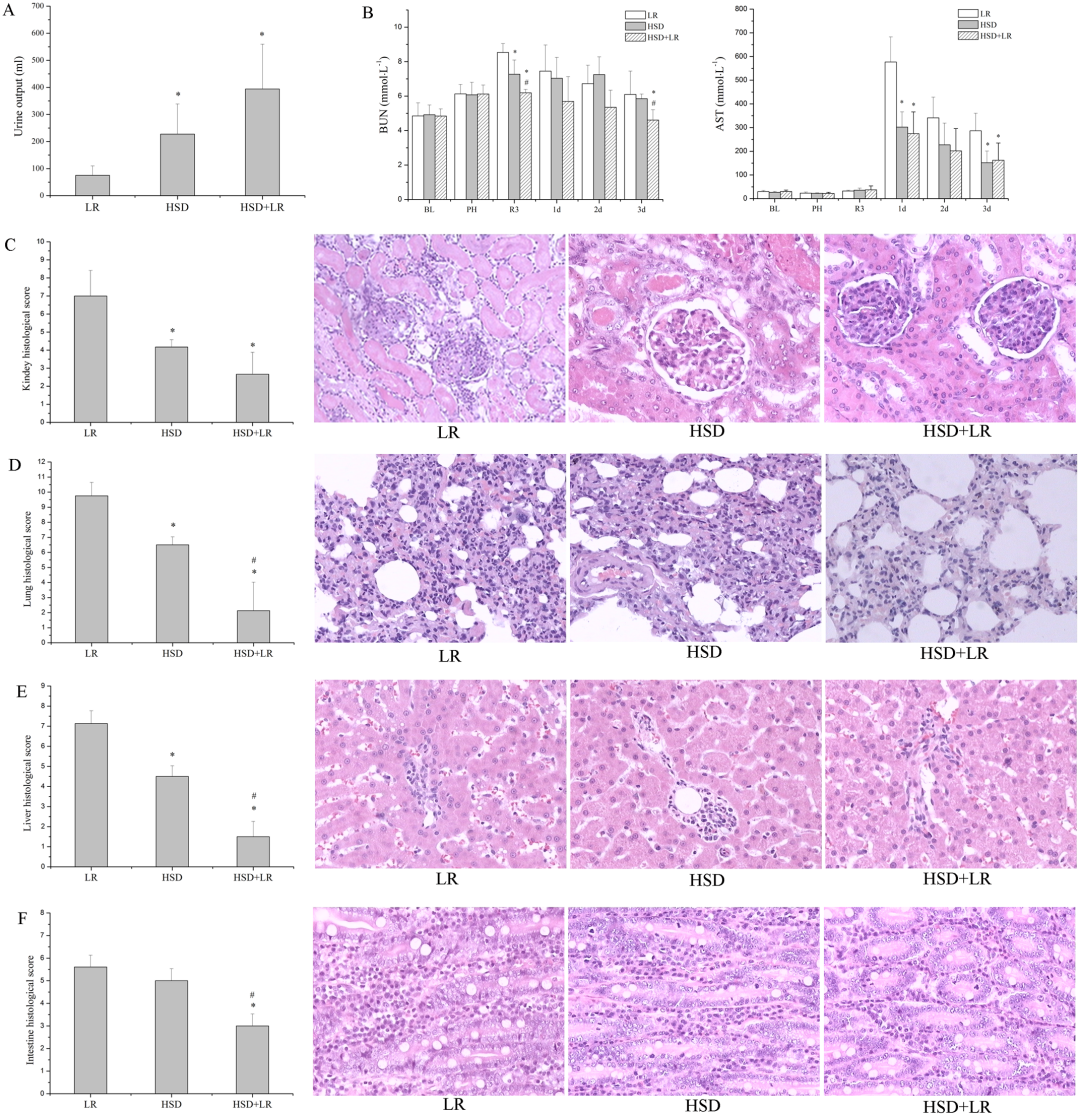
Effect of different HSD resuscitation strategies on organ damage. (A) The total urine output during the anesthetic period. (B) Plasma levels of urea nitrogen (BUN) and alanine aminotransferase (AST) measured at baseline (BL, time = -90 min), end of controlled bleeding (post-hemorrhage, PH, time = 0 min), 3 h after in-hospital resuscitation (Resuscitation3, R3, time = 240 min), and after one to three days of resuscitation (1d, 2d, 3d); After three days of resuscitation, the dogs were sacrificed and their tissues including kidney, lung, liver and intestinal were obtained for histological analysis. (C) Kidney histologic scores and appearance. (D) Lung histologic scores and appearance. (E) Liver histologic scores and appearance. (F) Intestinal histologic scores and appearance. H&E staining and Periodic Acid-Schiff staining for organs. original magnification, 200×. *P<0.05 vs LR; ^#^P<0.05 vs HSD. n = 6 for all groups.

The total urine output during the anesthetic period was significantly lower in the LR group than in the other groups (p < 0.05) (Fig 6A).

As shown in (Fig 6B), the hemorrhage and resuscitation caused a continual increase in the plasma levels of BUN. After blood transfusion, the BUN level gradually declined over the subsequent three days. The BUN value in the LR group was found to be significantly higher than the other groups at R3 (p < 0.05). Compared with BUN, the ALT level showed a different trend as time progressed: it seemed to increase before blood transfusion but then rapidly increased after 24 h (1 day, 1d) of resuscitation. The ALT value was significantly higher in the LR group than that in the other groups after 3 d of resuscitation (p < 0.05).

An increase in organ damage was confirmed by the increase in the total histologic score. The histologic score of the kidneys, lungs and liver were significantly lower in the HSD group than in the LR group (p < 0.05). The histologic score of the lungs, liver and Intestines were significantly lower in the HSD+LR group than in the HSD group (p < 0.05) (Fig 6C, 6D, 6E and 6F).

Histopathologic changes in the kidneys (Fig 6C): in the LR group, the structural integrity was shown to be marginally damaged, indicating mediate kidney vascular congestion. Many of the glomeruli were marginally degenerated and necrotized. Severe kidney tubular injury was illustrated by tubular cell swelling and necrosis, and interstitial inflammation was prominent. In the HSD group, the structures of the glomeruli were essentially intact, although local necrosis and minimal inflammatory cell infiltration was found in the kidney tubular epithelial cells. In the HSD+LR group, the structures of the glomeruli were relatively normal with the only abnormality being swollen kidney tubular epithelial cells. No significant interstitial inflammation was detected.

Histopathologic changes in the lungs (Fig 6D): the LR group developed more serious damage in the lungs, as identified by the infiltration of inflammatory cells throughout the lungs, moderate hyperplasia of the fibroblasts, significant fibrin exudation in alveolar cavity, and local bronchial epithelial cell deformation. Compared to the LR group, the HSD group showed mild damage with scattered inflammatory cell infiltration in the pulmonary interstitial space and no bronchial epithelial cell injury. For the HSD+ LR group, tissue damage was mild with a few inflammatory cell infiltrations, mild hyperplasia of the fibroblasts and a small amount of fibrin exudation.

Histopathologic changes in the liver (Fig 6E): In the LR group, the structure of the hepatic lobule was marginally broken, and cell swelling and vacuolar degeneration were found in the portal area. Marked inflammatory cell infiltration was present in the local area. Compared to the LR group, the structures of hepatic lobules in the HSD group were essentially intact, and the number of necrotic cells was lower. For the HSD+LR group, all these phenomena were attenuated.

Intestinal histopathologic changes (Fig 6F): interstitial inflammation and intestinal mucosa epithelial cell necrosis were found to be prevalent in the LR group. Cell necrosis was less pronounced in the HSD group than in the LR group, and the HSD+LR group revealed mild inflammation and necrosis.

## Discussion

In this beagle model of controlled hemorrhagic shock, the major findings of this study include the following. (1) Resuscitation after hemorrhagic shock with a bolus of HSD has a similar hemodynamic response as LR at ten times the volume of HSD, but HSD shows superior efficacy in organ protection, which suggests that the superior organ protection of HSD compared with LR is related to the fluid effect rather than the volume administered. (2) Although resuscitation after hemorrhagic shock with the combination of HSD and LR in the pre-hospital setting shows an increased risk of lung water, the combination of HSD and LR exhibit superior hemodynamic restoration, systemic oxygen metabolism and organ protection compared with HSD or LR used alone.

A fixed-pressure hemorrhage model according to Wiggers method was used in this study. The hypotensive shock state was maintained for 1h to achieve stable and serious physiological state. Fluid resuscitation was carried out for two periods. The initial fluid resuscitation occurred in the pre-hospital period for 1 h, spanning the time from hemorrhage to emergency medical services (EMS) [23, 24]. Subsequently, the fluid therapy was performed in the EMS or hospital facility until the operation or blood was prepared. The dose of HSD in a clinical rescue is typically 4 ml/kg, and previous animal studies showed that the hemodynamic benefits of an initial bolus of HSD (4 ml/kg) are similar to those observed after LR infusion at eight to ten times the volume of HSD [7, 15–17,]. In the proposed model of controlled hemorrhage, after resuscitation with HSD (4 ml/kg) or LR (40 ml/kg) in the pre-hospital setting, the MAP similarly increased to 75% of the baseline value. After the LR infusion in the hospital improved the MAP to 85% of the baseline value, the hemodynamic response was similar between the HSD group and the LR group. Then, the influence on tissue hypoxia and organ damage can be compared to show a similar hemodynamic efficacy; this difference is most likely related to the fluid effect rather than the volume administered.

From the results of systemic oxygenation, the HSD group shows similar DO_2_, VO_2_, ScvO_2_ and Lac but not P(v-a)CO_2_ compared to the LR group. The P_(v-a)_CO_2_ value in the HSD group was found to be significantly lower than that in the LR group at R3. Studies have reported that high venous-to-arterial differences in PCO_2_ (P_(v-a)_CO_2_) are related to low flow states, and P(v-a)CO_2_ may be considered a marker of the global hemodynamic status [25–28]. The better tissue perfusion observed in the HSD group compared to that in the LR group at R3 could have been due to the mobilization of water from the endothelium caused by the HSD; this likely increased the capillary luminal diameter by shrinking endothelial cells and improving microcirculatory flow [29, 30]. In addition, Zhao L [31] showed that HSD could improve RBC deformability and elevate plasma viscosity compared with an isotonic crystalloid resuscitation, which could be beneficial to maintain microcirculation.

The goal of fluid resuscitation is to alleviate cellular and tissue injury. Because organ and cellular dysfunction or injury can progress over time, long-term organ restoration is critical to evaluate the resuscitation effect. Therefore, in this study, the hisopathological damage in the kidneys, lungs, livers and intestines were evaluated after three days of resuscitation from hemorrhagic shock. Although resuscitation with HSD or LR alone produced similar hemodynamic and systemic oxygenation performances, HSD showed an advantage in organ protection compared with LR due to the observed higher urine outputs, lower plasma ALT levels and slighter histological changes. This could be explained by several reasons, which are described as follows. (1) Although the HSD group and LR group showed similar hemodynamic performances at the end of pre-hospital resuscitation, HSD produced a quicker increase in the MAP and CI than LR; this could produce better organ protection (Fig 4). (2) The experimental and clinical data suggest that hypertonic solutions produce anti-inflammatory [32, 33] and immunomodulatory [34, 35] effects, which might beneficially affect postresuscitation recovery and possibly decrease the rate of organ failure [5, 36, 37]. Activated neutrophils play a pivotal role in resuscitation injury. The types of fluids can affect the degree of neutrophil activation.LR resuscitation has been shown to produce an up-regulation of various neutrophil and endothelial adhesion molecules in models of hemorrhagic shock [38–40]. In contrast, hypertonic fluids have been shown to suppress neutrophil responses in vitro and to ameliorate neutrophil-mediated host tissue damage in animal and clinical studies [41–43]. While, HSD resuscitation resulted in the transient inhibition of PMN CD11b expression and partial restoration of the normal monocyte phenotype shortly after injury [32, 34]. Additionally, HSD significantly reduced the number of leukocytes accumulated in the liver after resuscitation of hemorrhagic shock [44]. In this study, the inflammatory cell infiltration in tissue has been assessed by histological analysis. Compared with LR, HSD resuscitation induced minor inflammatory injury in live and lung tissue (data not shown). (3) Data have suggested that fluids exert micro-circulatory effects independent of the volume expansion or oxygen-carrying capacity [45–47]. HSD may enhance the microcirculatory flow by drawing fluid from the edematous endothelium, resulting in a more significant benefit in re-establishing microvascular perfusion than standard crystalloids [48–50]. (4) Considering the kidneys, HSD resuscitation resulted in a greater urine output than LR, which could be partly due to the diuresis of HSD [51–54]. The reasons and mechanisms for the effects of HSD on organ protection should be studied in more detail.

From a previous animal study, only one report of fluid resuscitation used a combination of HSD and LR in an uncontrolled hemorrhage model [55]. In this study, an initial bolus of HSD with a continued infusion of Lactated Ringer appeared to be beneficial, in that it produced a better volume expansion than the results from using HSD or LR resuscitation alone. However, that study was limited due to a short observation time of only 60 min. In this study, fluid resuscitation with the combination of HSD and LR was designed to mimic the clinical procedure. A bolus of HSD was injected first and subsequently followed by LR outside of the hospital. Then, LR was continually transfused until the MAP reached 85% of the baseline value. As a result, although the MAP in the HSD+LR group was not different from that of the HSD and LR groups throughout the observation period, the hemodynamic parameters including CI, SVI, GEDVI and ITBVI in the HSD+LR group had significantly higher values than the other groups, which suggested that the effects of volume expansion in the HSD+LR group was nearly twice that of the other groups. However, higher Cl and MAP in pre-hospital period are associated with higher blood loss and mortality in an uncontrolled hemorrhagic shock. Moreover, the higher volume expansion induced stronger hydrostatic pressure in lung, which ultimately increased lung water and the risk of harmful effect on the respiratory function. In this study, resuscitation with total volume of HSD plus LR caused higher lung water and lower PaO_2_, but the value of PaO_2_ was still within the normal range, suggesting the volume used in this study was inadequate to result in serious lung damage. In addition, the improvement of hemodynamic and hypoxia in HSD+LR group ultimately induced lower grade lung histopathologic changes than HSD or LR alone.

Oxygen delivery (DO_2_), which is a function of hemoglobin (Hb), arterial oxygen saturation (SaO_2_), and cardiac index (CI), can be represented mathematically as DO_2_ = CI × 13.4 × Hb × SaO_2_. Following hemorrhage and fluid resuscitation, the Hb concentration may decline precipitously. However, unless pulmonary injury or respiratory embarrassment has occurred, gas exchange is usually not severely impaired, and the SaO_2_ remains normal. Therefore, the DO_2_ is mainly dependent on the CI level and Hb concentration. Generally, the effective volume expansion could theoretically lead to a state of relative hemodilution, producing a comparatively lower Hb concentration. Conversely, the effective volume expansion could result in higher cardiac output. In this study, the Hb concentration was in fact lower (Fig 5F), but the Cl level was significantly higher in the HSD+LR group than other groups (Fig 3A). Ultimately, the DO_2_ level was higher in the HSD+LR group than HSD or LR alone. ScvO_2_ reflects the balance between oxygen delivery and demand. It would decrease when oxygen delivery has been compromised or the systemic oxygen demands have exceeded the supply. ScvO_2_ can be represented mathematically as ScvO_2_ = SaO_2_- [VO_2_÷(1. 34×CO×Hb)]. Therefore, the value of ScvO_2_ was also associated with the CI level and Hb concentration. On the whole, the improvement in tissue hypoxia after resuscitation was not significant compared with the hemodynamic restoration. Resuscitation with the combination of HSD and LR showed better organ protection than HSD or LR alone.

This study has several limitations. First, the animal model of controlled hemorrhage does not reflect typically associated traumatic injuries. Thus, it does not reflect the associated traumatic injuries which could influence systemic inflammation and subsequent organ injuries. Second, clinical treatments for patients with hemorrhagic shock are complex, and the trends of resuscitation are an ongoing complex process; However in this study, a strict protocol of fluid transfusion was followed with no additional interventions. Therefore, this study has not identified the best resuscitation strategy, but only adds to the literature highlighting the differences between fluids and the effects of fluid combination. Third, although resuscitation in the HSD group produced superior organ protection than in the LR group, the mechanism of this requires more study. Finally, there may be errors in the measurement of the hemodynamic parameters because of the limitation of PiCCO technique. Despite the ability to calibrate measurements, the primary limitation of PiCCO pulse contour analysis remains the potential for inaccuracy due to the multitude of input variables required and the assumptions inherent in the derived components of the equation. In addition, the measured value of global end-diastolic volume (GEDV) may be larger than the actual value when the blood volume was too low (for example, in the end of hemorrhage). All these limitations reduced the impact of our results on clinical practice.

## Conclusions

We conclude that the current experimental data from this study indicate that resuscitation after hemorrhagic shock with a bolus of HSD (4 ml/kg) has a similar hemodynamic response compared with that of LR at ten times the volume of HSD (40 ml/kg), but HSD shows superior efficacy in organ protection, which confirms that the superior organ protection of HSD is related to the fluid effect rather than the volume administered. Although animals receiving resuscitation with the combination of HSD (4 ml/kg) and LR (40 ml/kg) in the pre-hospital setting and LR in the hospital would produce an increased risk of higher lung water, which may ultimately worsen respiratory function, the combination of HSD and LR exhibits better hemodynamic restoration, superior systemic oxygen metabolism and organ protection compared with HSD or LR used alone. Thus, based on the results of this study, HSD could be recommended as the initial fluid used in ambulance rescues for treating hemorrhagic shock.
